# Synthesis of Vinyl-Containing MQ Copolymers in Active Medium

**DOI:** 10.3390/polym18030315

**Published:** 2026-01-24

**Authors:** Alina Khmelnitskaia, Aleksandra Kalinina, Ivan Meshkov, Ekaterina Ivanova, Sergey G. Vasil’ev, Alexander Buzin, Gagik Ghazaryan, Sergey Ponomarenko, Aziz Muzafarov

**Affiliations:** 1 Enikolopov Institute of Synthetic Polymeric Materials, Russian Academy of Sciences, Profsoyuznaya 70, 117393 Moscow, Russia; alina.khmelnitskaya@ispm.ru (A.K.); ivanbm@ispm.ru (I.M.); al37919@gmail.com (A.B.); gagik@ispm.ru (G.G.); ponomarenko@ispm.ru (S.P.); aziz@ispm.ru (A.M.); 2 Chemistry Department, Lomonosov Moscow State University, GSP-1, Leninskie Gory 1/3, 119991 Moscow, Russia; ekanerina.iva@gmail.com; 3 Federal Research Center of Problems of Chemical Physics and Medicinal Chemistry, Russian Academy of Sciences, Ac. Semenov Avenue 1, Chernogolovka, 142432 Moscow, Russia; viesssw@mail.ru

**Keywords:** MQ copolymers, vinyl-containing siloxanes, active medium, hydrolytic polycondensation, molecular composites

## Abstract

MQ copolymers, consisting of monofunctional (M) and tetrafunctional (Q) siloxane units, are versatile materials used as additives, adhesives, and in composite materials. Functional groups, such as vinyl substituents, in M-units allow for the tailoring of properties for specific applications. This study aimed to synthesize vinyl-containing MQ copolymers (M^Vin^MQ) via a controlled, chlorine-free method to explore the regulation of their composition and properties. The results demonstrated precise control over the copolymer architecture, with hydroxyl content and molecular weight increasing alongside the Q-unit fraction. All obtained copolymers exhibited high thermal stability, with 5% mass loss occurring above 295 °C in air and 365 °C in argon. Fractionation data supported a molecular composite model consisting of an inorganic core and an organic shell. Polycondensation in an active medium is an effective method for the directed synthesis of structurally tunable M^Vin^MQ copolymers, offering a versatile platform for developing functional hybrid materials, modifiers, and cross-linking agents.

## 1. Introduction

By their structure, MQ copolymers are compounds composed of monofunctional siloxane units ([R_3_SiO_0.5_]) (M-units) and tetrafunctional units ([SiO_4/2_]) (Q-units) [1,2]. These copolymers have a wide range of applications due to their versatility. They are used as fillers [3], reinforcing additives [4,5,6,7,8], pressure-sensitive adhesive components [9,10], and cross-linking catalysts [11], and are in high demand in various other industries [12,13,14,15,16]. This diversity of applications is attributed to the properties of MQ copolymers, such as the simplicity of their synthesis and the lack of insoluble structures even after prolonged condensation at high temperatures.

It has been shown in [17,18] that MQ copolymers are molecular composites composed of several components: high-molecular-weight components act as a filler, medium components act as a polymer matrix, and low-molecular-weight components act as plasticizers. At the same time, all components are mixed together without any limitations.

Changing the organic substituent in a triorganosiloxane unit opens up wide possibilities for improving the properties of MQ copolymers for specific applications [19,20,21]. In addition to hydroxyl groups formed during synthesis, other functional groups can be introduced into M-units through various methods. Significant changes in the properties also occur when the structure of an M-unit is modified or when methyl substituents are replaced with aryl substituents like phenyl groups [22].

Currently, the most efficient method for producing MQ-type copolymers is polycondensation of alkoxysilanes in an “active medium”, which is an excess of anhydrous acetic acid. The acid acts not only as a reactant but also as a solvent and catalyst during the reaction [23]. The mechanism of this process can be described as a series of sequential reactions in which water is produced during the esterification of acetic acid with alcohol and consumed during the subsequent hydrolysis of acetoxy derivatives [17]. This rapid exchange of water into the reaction prevents phase separation, ensuring uniformity in the reaction mixture.

Thus, in addition to MQ copolymers with methyl substituents [24], MQ copolymers with phenyl [22], sydnone [25], and decyl [26,27] substituents were successfully obtained by polycondensation in an “active medium”. Thus, this method provides a versatile framework for developing a practical combinatorial platform.

In fact, the synthesis of MQ copolymers has an almost endless number of variations in terms of methods and processes. These compounds are “core-shell” systems that, during synthesis, transform into molecular composites consisting of several components that mix together without any restrictions [17,28]. There is a high-molecular-weight fraction that acts as a filler, a medium-weight fraction acting as a polymer matrix, and a low-molecular-weight fraction that works as a plasticizer.

Introducing a vinyl group into the structure of the product opens up possibilities for its further modification by, for instance, thiolene reactions [29,30,31].

Therefore, the use of the term “resin” to describe these objects, which are synthetic oligomers that harden to form non-melting and insoluble products, is not appropriate. The main difference between the traditional approach and the novel method is the ability to control the properties of the copolymers by controlling their structure. This allows for more precise and targeted control of the composition and properties of the materials, rather than relying on an empirical approach.

The new method also provides a better understanding of the underlying mechanisms behind the complex transformations that take place during the formation of siloxane bonds. While the potential of this approach in the synthesis of copolymers with functional vinyl groups has not yet been fully explored, there are numerous examples of its practical applications in various industries. The relevance of developing modern chlorine-free methods for the controlled synthesis of these objects is determined and reflected in the literature [32,33,34,35,36,37].

The purpose of this study is to synthesize vinyl-containing copolymers of M^Vin^MQ, where M^Vin^ represents vinyldimethylsilyl units, M represents trimethylsilyl units, and Q represents tetrafunctional units of silicon.

The synthesis was carried out using the method of hydrolytic polycondensation in an active medium. The structure of these copolymers was analyzed and the possibilities of controlling the content of vinyl groups and the ratio of M- and Q-units were explored.

## 2. Materials and Methods

### 2.1. Materials

Unless otherwise stated, all chemicals were reagent-grade and used as received without purification.

Tetraethoxysilane (TEOS), 1,1,3,3-Tetramethyl-1,3-divinyldisiloxane (TMDVDS), hexamethyldisiloxane (HMDS), methyl tertbutyl ether (MTBE), acetic acid anhydrous sodium sulfate, pyridine, and toluene were purchased from ECOS-1 (Moscow, Russia); trimethylchlorosilane 97% and acetyl chloride were purchased from ABCR (Karlsruhe, Germany).

Trimethylchlorosilane was distilled under a stream of argon immediately before the reaction.

All reagents were purified by standard methods [38]. Before the synthesis, acetic acid was dried over P_2_O_5_, distilled, and kept in an inert atmosphere. Pyridine was dried over BaO, distilled, and kept under 3Å molecular sieves.

### 2.2. Characterization

^1^H nuclear magnetic resonance (NMR) spectra were recorded at room temperature on a Bruker WP 250 SY spectrometer (250.13 MHz) (Bruker, Eltingen, Germany).

^29^Si NMR spectra were recorded on a Bruker Avance III 400 spectrometer (Bruker, Eltingen, Germany). Analysis was performed using a solid-state probe under magic-angle spinning with the frequency of 8 kHz, cross-polarization, and ^1^H decoupling. The spectra were processed using MestReNova software (v. 12.0).

IR spectra were recorded on an IR Fourier spectrometer—Nicolet iS50 (Thermo Scientific, Waltham, MA, USA)—in the ATR mode with 32 scans for each wave number in the range of 1000–4000 cm^−1.^

Gel permeation chromatograms (GPCs) were recorded using a GPC system, consisting of high-pressure pump Stayer Series II (Akvilon, Podolsk, Russia), refractometric detector RIDK 102 (Laboratory Instruments Prague, Prague, Czech Republic), and column thermostat JETSTREAM 2 PLUS (KNAUER, Berlin, Germany). With the temperature controlled at 40 °C, toluene was used as the eluent, with a flow rate 1 mL/min. Analytical separation was performed using a 7.8 mm × 300 mm Phenomenex column (Spectralab Scientific Inc., Torrance, MA, USA) filled with Phenogel sorbent with a pore size ranging from 500 Å to 104 Å. Molecular masses were determined using polystyrene standards.

The samples were analyzed using DSC on a DSC-822e differential scanning calorimeter (Mettler Toledo, Schwerzenbach, Switzerland) at a heating rate of 10 °C/min.

Thermogravimetric studies were performed using a DerivatographC (MOM, Budapest, Hungary) at a heating rate of 5 °C/min in air and under an argon atmosphere on samples weighing approximately 10 mg.

Preparative chromatography for purification consisted of a Shimadzu LC-20AT high-pressure pump (Tokyo, Japan), a RIDK-102 refractometric detector (Prague, Czech Republic), and 300 × 21.2 mm preparative columns packed with Phenogel sorbent (Phenomenex, Torrance, California, USA) with a particle size of 10 μm. Depending on the molecular weights of the components of the mixtures being separated, columns with pore sizes of 103 Å, 104 Å, and 105 Å were used. The eluent was THF, at room temperature and a flow rate of 10 mL/min.

Elemental analysis was performed on a Carlo Erba 1106 instrument (Milan, Italy). The relative error in determining silicon, carbon, and hydrogen contents did not exceed 0.1 wt%. Silicon, carbon, and hydrogen contents were determined by combustion of a sample (5 × 10 × 10^–3^ g) in an oxygen atmosphere at a temperature of 950 °C.

The intrinsic viscosity of the polymers was measured using a Schott capillary viscometer (Ubbelohde No. 531 01, Mainz, Germany) with a capillary diameter of 0.53 mm and a capillary viscometer (No. 537 10, Mainz, Germany) with a capillary diameter of 0.63 mm. Measurements were performed at 25 °C with a temperature calibration accuracy of 0.1 °C. The universal calibration method was used to determine the hydrodynamic radii (R) and viscosity-average molecular weights (M_η_).

### 2.3. General Method for Obtaining Vinyl-Containing MQ Copolymers with an M/Q Ratio of 1/2

In a 100 mL two-necked round-bottomed flask equipped with a magnetic stirrer, re-flux condenser, and calcium chloride tube, 10.7 mL of tetraethoxysilane (0.048 mol), 2.1 mL of 1,1,3,3-tetramethyl-1,3-divinyldisiloxane (9 mmol), 0.64 mL of hexamethyldisiloxane (3 mmol), 34.3 mL of acetic acid (0.6 mol), and 0.045 mL of acetyl chloride (0.5 wt.% from tetraethoxysilane) were added. The reaction mixture was refluxed for 6 h at 118 °C. The product was washed with water until the washings were neutral and kept over anhydrous sodium sulfate for 12 h. The mixture was then filtered, and volatile compounds were removed on a rotary evaporator. The yield of the products was 90–96%.

M^Vin^MQ-25: ^1^H NMR (250 MHz, acetone-d_6_) δ, ppm: 5.71–6.39 (m, 1H), 0.19–−0.58 (m, 7H). GPC (toluene, 20 kDa, PSS): M_p_ = 1900, M_n_ = 1700, M_w_ = 4800, M_w_/M_n_ = 2.8. Found (%): C, 20.5; H, 4.8; Si, 38.8. Calculated (%): C, 20.7; H, 4.9; Si, 40.3.

M^Vin^MQ-50: ^1^H NMR (250 MHz, acetone-d_6_) δ, ppm: 5.68–6.37 (m, 3H), 0.23 (d, J = 15.2 Hz, 15H). GPC (toluene, 20 kDa, PSS): M_p_ = 1800, M_n_ = 1100, M_w_ = 3200, M_w_/M_n_ = 2.9. Found (%): C, 22.3; H, 5.0; Si, 39.1. Calculated (%): C, 22.5; H, 5.0; Si, 39.0.

M^Vin^MQ-75: ^1^H NMR (250 MHz, acetone-d_6_) δ, ppm: 5.68–6.46 (m, 2H), 0.22 (d, J = 15.2 Hz, 7H). GPC (toluene, 20 kDa, PSS): M_p_ = 1800, M_n_ = 1100, M_w_ = 3000, M_w_/M_n_ = 2.7. Found (%): C, 23.0; H, 4.7; Si, 37.2. Calculated (%): C, 22.3; H, 4.9; Si, 38.4.

M^Vin^MQ-100: ^1^H NMR (250 MHz, acetone-d_6_) δ, ppm: 5.68–6.41 (m, 3H), 0.26 (s, 6H). GPC (toluene, 20 kDa, PSS): M_p_ = 1300, M_n_ = 1300, M_w_ = 2900, M_w_/M_n_ = 2.2. Found (%): C, 22.9; H, 4.9; Si, 37.5. Calculated (%): C, 23.0; H, 4.6; Si, 38.2.

### 2.4. Method for Obtaining Vinyl-Containing MQ Copolymers with an M:Q Ratio of 1:1

In a 250 mL two-neck round-bottomed flask equipped with a magnetic stirrer, reflux condenser, and calcium chloride tube, 22.2 mL of tetraethoxysilane (0.1 mol), 5.8 mL of 1,1,3,3-tetramethyl-1,3-divinyldisiloxane (0.025 mol), 5.3 mL of hexamethyldisiloxane (0.025 mol), 65.8 mL of acetic acid (1.15 mol), and 0.094 mL of acetyl chloride (0.5 wt.% from tetraethoxysilane) were added. The reaction mixture was refluxed for 6 h at 118 °C. The product was washed with water until the washings were neutral and kept over anhydrous sodium sulfate for 12 h. Then it was filtered and volatile compounds were removed on a rotary evaporator. As a result, 4.8 g of the product was obtained (yield 92%). ^1^H NMR (250 MHz, CDCl_3_) δ, ppm: δ 5.40–6.42 (m, 3H), −0.28–0.62 (m, 16H). GPC (toluene, 20 kDa, PSS): M_p_ = 1100, M_n_ = 1500, M_w_ = 2600, M_w_/M_n_ = 1.7. Found (%): C, 28.6; H, 6.1; Si, 37.9. Calculated (%): C, 28.6; H, 6.1; Si, 38.1.

### 2.5. Method for Obtaining Vinyl-Containing MQ Copolymers with an M:Q Ratio of 1:3

To a 100 mL two-necked round-bottomed flask equipped with a magnetic stirrer, reflux condenser, and calcium chloride tube, 11.1 mL of tetraethoxysilane (0.05 mol), 1.0 mL of 1,1,3,3-tetramethyl-1,3-divinyldisiloxane (4.25 mmol), 0.9 mL of hexamethyldisiloxane (4.25 mmol), 26.4 mL of acetic acid (0.625 mol), and 0.47 mL of acetyl chloride (0.5% from tetraethoxysilane) were added. The reaction mixture was refluxed for 6 h at 118 °C. The product was washed with water until the washings were neutral and kept over anhydrous sodium sulfate for 12 h. Then it was filtered and volatile compounds were removed on a rotary evaporator. As a result, 1.0 g of the product was obtained (yield 90%). ^1^H NMR (250 MHz, acetone-d_6_) δ, ppm: δ 6.18 (s, 1H), 6.00 (s, 1H), 5.87 (s, 1H), 0.21 (d, J = 17.3 Hz, 18H). GPC (THF, 1000 kDa, PSS): M_p_ = 10,900, M_n_ = 16,300, M_w_ = 80,400, M_w_/M_n_ = 4.9. Found (%): C, 17.5; H, 4.1; Si, 38.1. Calculated (%): C, 15.7; H, 3.4; Si, 41.9.

### 2.6. Blocking of MQ Copolymers

A 10 wt. % solution of M^Vin^MQ-X (1.0 g, 0.0048 mol) in MTBE, trimethylchlorosilane (2.67 g, 0.025 mol), and pyridine (1.93 g, 0.025 mol) were added to a 20 mL round-bottom flask. The reaction mixture was boiled with constant stirring for 2 h. The product was washed with water until the washings were neutral and kept over anhydrous sodium sulfate for 12 h. Then it was filtered and volatile compounds were removed on a rotary evaporator. ^1^H NMR (250 MHz, CDCl_3_) δ, ppm: 6.05 (ddd, J = 53.6, 36.9, 18.5 Hz, 2H), 0.12–0.67 (m, 12H). IR 2955 cm^−1^ ν_as_ (CH_3_-), 2923 cm^−1^ ν_as_ (-CH_2_-), 1467 cm^−1^ ν_um_ (-CH_2_-), 1260 cm^−1^ ν (Si-CH_3_).

## 3. Results and Discussion

**Figure 1:**
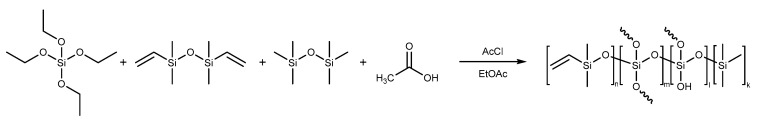
General scheme for the synthesis of M^Vin^MQ copolymers.

The synthesis of M^Vin^MQ copolymers was carried out by polycondensation of tetraethoxysilane with an excess of acetic acid, using hexamethyldisiloxane and 1,3-divinyl-1,1,3,3-tetramethyldisiloxane as precursors for triorganosilyl units, in the presence of catalytic amounts of acetyl chloride (Figure 1).

By varying the amounts of the initial reagents used in the polycondensation process, the proportion of M^Vin^-units was varied from 0.25 to 1 relative to the total number of M-type units, and the ratio of M- and Q-type units was varied from 1:1 to 1:3. The ratio of M and Q-units significantly influences characteristics such as molecular weight and silanol content. Changing the M/Q ratio from 1 to 0.33 changes the physical state of MQ copolymers from viscous liquids to soluble solid powders. Increasing the M-component content in the initial reaction mixture reduces the residual silanol content in the final product [9].

**Figure 2:**
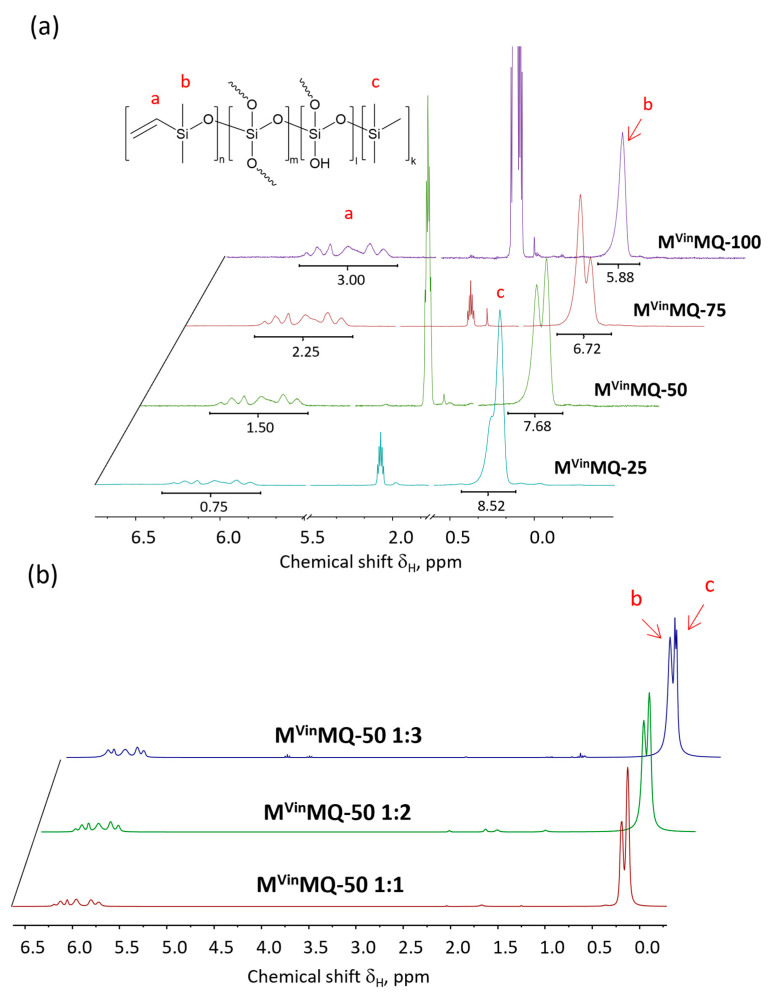
^1^H NMR spectrum of (**a**) M^Vin^MQ copolymers with different ratios of functional groups, (**b**) M^Vin^MQ-50 1:1, 1:2, 1:3, where a—protons of the vinyl group of the terminal dimethylvinylsilyl unit, b—protons of the methyl group of the dimethylvinylsilyl unit, and c—protons of the methyl group of the trimethylsilyl unit.

Thus, copolymers of the general formula M^Vin^MQ were synthesized with a ratio of M- and Q-units equal to 1:2, and the ratio of M^Vin^-units to M-units equal to 100/0, 75/25, 50/50, and 25/75, respectively. Additionally, we synthesized a ratio of M- and Q-units of 1 to 1 and 1 to 3 with a ratio of M^Vin^-units to M-units of 50/50. To indicate the number of functional groups in the copolymer, the designation M^Vin^MQ-X was introduced, where X represents the number of vinyl groups. The process was monitored using ^1^H NMR spectroscopy by the disappearance of proton signals from alkoxy groups (Figure S1 in the Supplementary Materials). Figure 2 shows the ^1^H NMR spectra of the polycondensation reaction products. The signals in the range of 5.77–6.07 ppm correspond to the protons of the vinyl group in the vinyl dimethylsilyl unit, and the signals with δ_H_ = 0.12 and 0.35 ppm belong to the protons of methyl groups in the vinyl dimethylsilyl and trimethylsilyl units, respectively. The ratio of the integral signal intensities of protons of the vinyl group to protons of the methyl group after the polycondensation reaction in an active medium corresponds to a preset one. The total conversion time of the alkoxy groups was 8 h.

Analysis of the structure of the copolymers obtained was conducted using two methods. The first involved the study of M^Vin^MQ copolymers directly produced in an active medium using ^29^Si NMR solid-state spectroscopy. The second method was a more traditional approach for analyzing such structures obtained in an active medium and involved using a combination of data from elemental analysis of the polycondensation product and the number of hydroxyl groups calculated from proton NMR spectroscopy of the samples after they had been pre-blocked with monochlorosilane.

The study of the structure of M^Vin^MQ copolymers using ^29^Si NMR solid-state spectroscopy allowed us to determine the structural composition of the products obtained. The results are summarized in Table 1.

**Figure 3:**
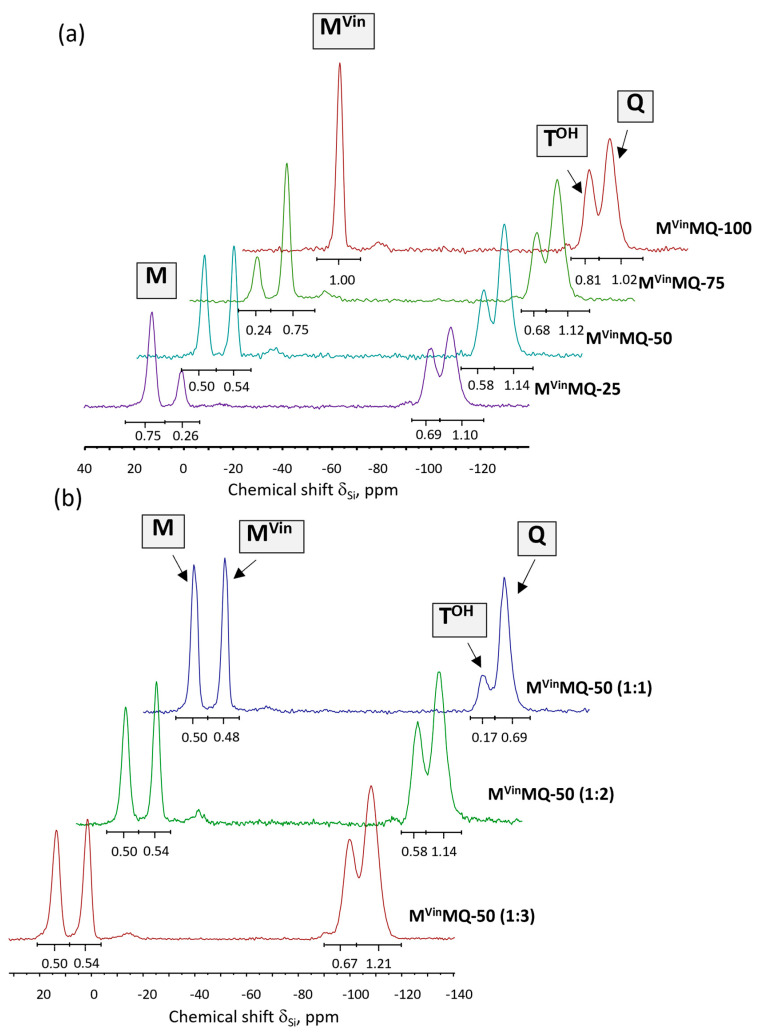
^29^Si solid-state NMR spectra of (**a**) M^Vin^MQ copolymers 1:2, (**b**) M^Vin^MQ-50 1:1, 1:2, 1:3.

The ^29^Si NMR spectra of M^Vin^MQ copolymers (Figure 3) show signals at 15 ppm corresponding to the terminal trimethylsilyl groups (M-units), signals at 0 ppm corresponding to the terminal vinyl dimethylsiloxy groups (M^Vin^-units), and signals between −100 and −110 ppm corresponding to the silsesquioxane analog of the tetrafunctional Q-unit with one hydroxyl group (T^OH^-units) and the SiO_4/2_ unit (Q-units), respectively. The reactivity of M^Vin^MQ copolymers increases with Q-reagent content, as this raises the number of hydroxyl groups. Consequently, while M^Vin^MQ copolymers with a 1:1 ratio are stable in their neat form, a copolymer with a 1:3 ratio must be stored in solution to maintain stability.

The ratio of integrated signal intensities for different types of silicon atoms corresponds to the theoretical structure calculated based on the amount of reagents used, both in terms of M- and M^Vin^-units and in terms of total number of M-units and T^O-^ and Q-units, regardless of their initial ratios.

Thus, the ^29^Si NMR solid-state spectroscopy data confirm that the ratio of trimethylsilyl and vinyldimethylsilyl groups and M- and Q-type units in the composition of copolymers corresponds to the calculated ratio.

**Figure 4:**
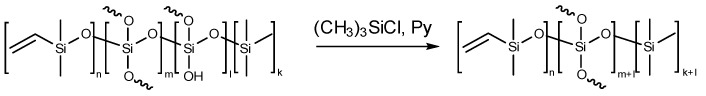
General scheme of blocking of M^Vin^MQ copolymers.

To quantify the hydroxyl groups in the structure of the MQ-copolymer, they were blocked with trimethylchlorosilane (Figure 4).

**Figure 5:**
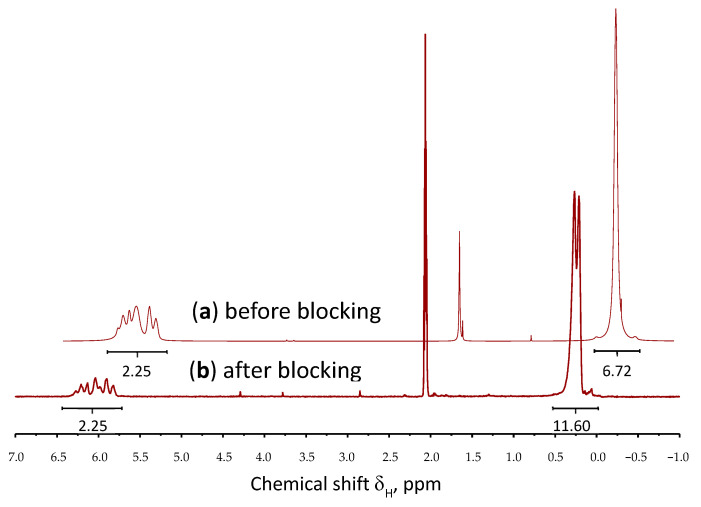
^1^H NMR spectra of M^Vin^MQ copolymers before (**a**) and after (**b**) blocking.

The correlation of the integrated proton intensities in the region of methyl groups at silicon atoms in the spectra before and after blocking allowed us to determine the content of hydroxyl groups (T^OH^-units) in the MQ copolymers ranging from 2.5% to 5.3% (Figure 5, Table 1).

**Figure 6:**
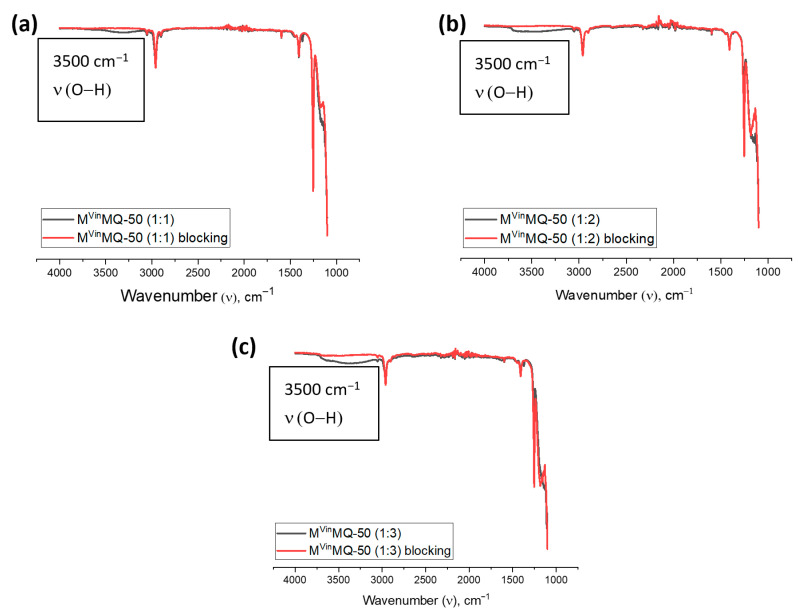
IR spectra before and after blocking of the copolymers: (**a**) M^Vin^MQ-50 (1:1); (**b**) M^Vin^MQ-50 (1:2); (**c**) M^Vin^MQ-50 (1:3).

The blocking efficiency was confirmed using IR spectroscopy by the disappearance of an absorption band in the region of 3500 cm^−1^ (Figure 6 and Figure S2 in the Supplementary Materials). According to IR spectroscopy data, it can be seen that, as the number of Q-units increases, so does the number of hydroxyl groups in the product—the absorption band intensity increases in the 3500 cm^−1^ region. These data correspond to the NMR spectroscopy measurements before and after blocking of the product.

The analysis of the chemical composition and structure of the MQ copolymers obtained was carried out based on the data from elemental analysis and NMR spectroscopy (Table 1). The error in determining the content of elements (silicon, carbon, hydrogen) did not exceed 0.1% of the measured value. According to the results presented in Table 1, the composition of all copolymers slightly differed from that calculated using ^29^Si NMR spectroscopy. Basically, excess values were observed for carbon, which may indicate a larger number of M-units at the end of the copolymer chain.

Thus, the structure of the MQ copolymers, as calculated from the blocking and elemental analysis data, coincides with the calculated values, and both methods of proving the structure correlate with each other.

**Figure 7:**
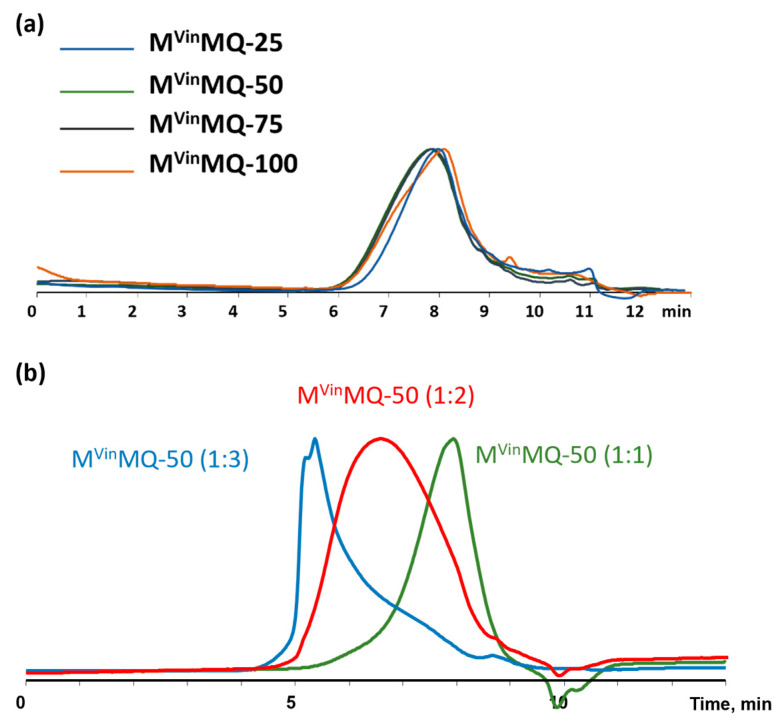
GPC curves of (**a**) M^Vin^MQ copolymers 1:2, (**b**) M^Vin^MQ-50 copolymers 1:1, 1:2, 1:3.

The molecular weight characteristics of the copolymers were determined using gel-permeation chromatography (GPC) (Figure 7).

According to the GPC data, one can see that, regardless of the ratio of M^Vin^-units to M-units, the MM of products are close to each other. All copolymers have a peak molecular weight of around 1800 and a molecular weight distribution (PDI) of around 2.7 (Table 1). As the number of Q-units increases, the molecular weight and polydispersity index also obviously increase (Table 1).

**Figure 8:**
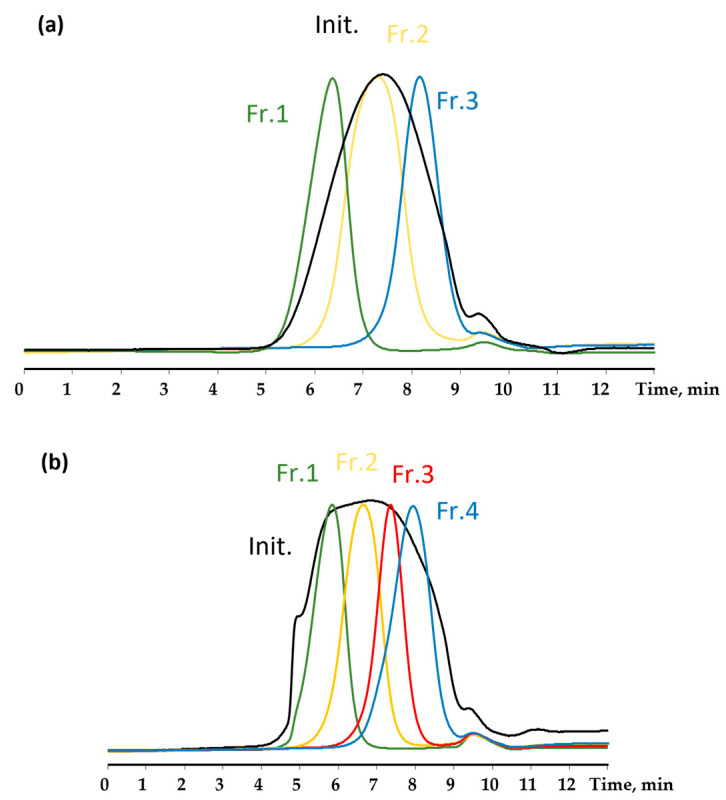
GPC curves of the copolymers (**a**) M^Vin^MQ-50 and (**b**) M^Vin^MQ-100.

The blocked copolymers, M^Vin^MQ-50 (1:2) and M^Vin^MQ-100 (1:2), were fractionated using preparative GPC (Figure 8).

Each fraction was analyzed using elemental analysis. The mass ratios of the fractions M^Vin^MQ-50 and M^Vin^MQ-100 were 17.5/55/27.5 and 18/39/21/22 mass units, respectively. The characteristics of all fractions are presented in Table 2, where [η] is intrinsic viscosity, R is the hydrodynamic radius, M_p_ is the molecular weight of the peak determined using a polystyrene standard, and M_η_ is the molecular weight of copolymers calculated using the universal calibration method.

The data obtained on the fractionation of copolymers and the determination of molecular weight characteristics correlate with the literature data on the composite nature of MQ copolymers [17].

When analyzing the results of the fractionation of all copolymers (Table 2), it was found that the elemental compositions of the high- and medium-molecular-weight fractions of M^Vin^MQ-50 and M^Vin^MQ-100 were similar, while the low-molecular-weight fraction contained more carbon and less silicon. This observation suggests that the low-molecular-weight fractions contained more terminal trimethyl- and dimethylvinylsilyl groups, while the higher-molecular-weight fractions have a higher proportion of the SiO_4/2_ core.

For M^Vin^MQ copolymers obtained, the values of the characteristic viscosity [η] were measured at a temperature of 25 °C. Table 2 shows that as the molecular weight of the polymer increases, so does the value of [η]. At the same time, the lower viscosity values indicate the more dense, globular shape of the macromolecules.

Using the GPC data and the values of [η] for toluene, the hydrodynamic radius R and the viscosity-average molecular weight, M_η_, of copolymers were calculated using the universal calibration method [39]. Table 2 shows the calculated values of these molecular characteristics compared to M_p_ (GPC).

Thus, it was demonstrated that polycondensation in the active medium allows for the regulation of the structure of M^Vin^MQ copolymers, as well as the content of vinyl functional groups and M- and Q-units.

**Figure 9:**
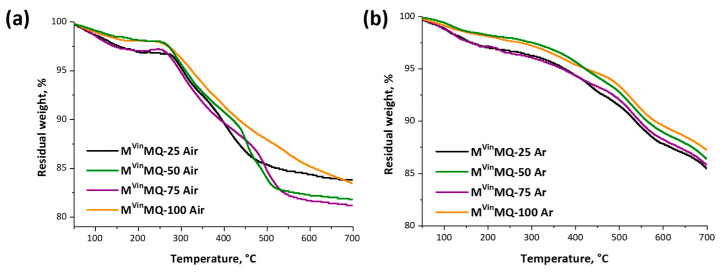
TGA curves in air (**a**) and argon (**b**) of MQ copolymers.

The thermal (in argon) and thermooxidation (in air) stability of the resulting MQ copolymers was evaluated using the TGA method (Figure 9).

Based on the TGA data, when heated in air and argon, we can conclude that the temperatures at which 5% mass loss occurs (T_5%_) for all samples are above 295 °C and 365 °C, respectively (Table 3). The M^Vin^MQ copolymers exhibit sufficiently high coke residue values at 700 °C (more than 80%).

The phase behavior of the products obtained was determined using differential scanning calorimetry (DSC) (see Figure S3 in the Supplementary Information). Based on the thermograms, no transitions were observed for any of the copolymers.

Thus, the study demonstrated that polycondensation in the active medium is a promising method for producing functional M^Vin^MQ copolymers. This allows for regulation of both the ratio of M- and Q-units, ranging from 1:1 to 1:3, as well as the number of vinyl groups. It was shown that the number of hydroxyl groups in the copolymers synthesized naturally increases with the increase in the content of Q-units in the composition. All copolymers exhibited thermal stability up to 295 °C and thermal oxidation resistance up to 365 °C. The possibility of controlled functionalization of M^Vin^MQ copolymers and their subsequent modification opens up new perspectives on the development of molecular composites, nanofluids, adhesives, thickening agents, and other applications.

## 4. Conclusions

In this work, the effectiveness of the polycondensation in the “active medium” for the directed synthesis of vinyl-containing MQ-copolymers (M^Vin^MQ) is demonstrated. The key advantage of using this approach is the possibility of precise control over the structure of the final product, namely, changing the ratio between the M- and Q-units (from 1:1 to 1:3) and functional vinyl groups in M-units.

It was found that as the proportion of Q-units increases, the content of hydroxyl groups and molecular weight of the products naturally increase. The obtained M^Vin^MQ copolymers exhibit high thermal and thermo-oxidative stability, beginning to degrade with a loss of 5% mass at temperatures above 295 °C in air and 365 °C in an inert atmosphere. The fractionation data and analysis of macromolecular characteristics of the obtained fractions support the model of M^Vin^MQ copolymers as molecular composites, with high-molecular-weight fractions forming an inorganic “core” and low-molecular substituent-rich “shells”.

The method developed is thus effective for producing functionalized M^Vin^MQ copolymers with specific properties, opening up wide opportunities for practical applications as modifiers, cross-linking agents, and key components in the development of new hybrid materials.

## Figures and Tables

**Table 1:** Composition and molecular weight characteristics of M^Vin^MQ copolymers.

Sample (M:Q)	M^Vin^:M:Q:T^OH^ from EA + ^1^H NMR	M^Vin^:M:Q:T^OH^ from ^29^Si NMR	Molecular Weight				OH, %	Elementary Analysis Experimental/Theoretical		
			M_n_	M_w_	M_p_	PDI		%C	%H	%Si
M^Vin^MQ-25 (1:2)	0.25:0.75:1.79:0.21	0.26:0.75:1.1:0.69	1700	4800	1900	2.8	4.2	$\frac{20.5}{20.7}$	$\frac{4.8}{4.9}$	$\frac{38.8}{40.3}$
M^Vin^MQ-50 (1:2)	0.5:0.5:1.6:0.4	0.54:0.5:1.14:0.58	1100	3200	1800	2.9	3.2	$\frac{22.3}{22.5}$	$\frac{5.0}{5.0}$	$\frac{39.1}{39.0}$
M^Vin^MQ-75 (1:2)	0.75:0.25:1.47:0.53	0.75:0.24:1.12:0.68	1100	3000	1800	2.7	4.2	$\frac{23.0}{22.3}$	$\frac{4.7}{4.9}$	$\frac{37.2}{38.4}$
M^Vin^MQ-100 (1:2)	1:0:1.45:0.55	1:0:1.02:0.81	1300	2900	1300	2.2	4.3	$\frac{22.9}{23.0}$	$\frac{4.9}{4.6}$	$\frac{37.5}{38.2}$
M^Vin^MQ-50 (1:1)	0.5:0.53:0.78:0.22	0.48:0.5:0.69:0.17	1500	2600	1100	1.7	2.5	$\frac{28.6}{28.6}$	$\frac{6.1}{6.1}$	$\frac{37.9}{38.1}$
M^Vin^MQ-50 (1:3)	0.5:0.5:2.15:0.85	0.5:0.5:1.21:0.67	16,300	80,400	10,900	4.9	5.3	$\frac{17.5}{15.7}$	$\frac{4.1}{3.4}$	$\frac{38.1}{41.9}$

**Table 2:** Properties of the fractions of copolymers M^Vin^MQ-50 and M^Vin^MQ-100.

M^Vin^MQ-X (1:2)	fr. №	Fraction Yield [%]	M_p_ (GPC)	[η] [dL/g] Toluene	R [nm]	M_η_	Elemental Analysis Expt/Theor		
							C [%]	H [%]	Si [%]
M^Vin^MQ-50	1	17.5	15,600	0.010	3.6	305,100	$\frac{23.5}{22.5}$	$\frac{5.4}{5.0}$	$\frac{38.5}{39.0}$
	2	55	6400	0.012	2.2	54,400	$\frac{23.3}{22.5}$	$\frac{5.5}{5.0}$	$\frac{38.7}{39.0}$
	3	27.5	2800	0.007	1.5	28,700	$\frac{25.7}{22.5}$	$\frac{6.1}{5.0}$	$\frac{37.3}{39.0}$
M^Vin^MQ-100	1	18	26,300	0.023	4.2	195,300	$\frac{23.4}{23.0}$	$\frac{5.0}{4.6}$	$\frac{38.4}{38.2}$
	2	39	12,400	0.018	3.2	116,400	$\frac{23.6}{23.0}$	$\frac{5.1}{4.6}$	$\frac{38.5}{38.2}$
	3	21	6000	0.009	2.2	80,100	$\frac{24.8}{23.0}$	$\frac{5.4}{4.6}$	$\frac{37.4}{38.2}$
	4	22	3500	0.008	1.6	34,500	$\frac{26.8}{23.0}$	$\frac{5.8}{4.6}$	$\frac{37.1}{38.2}$

**Table 3:** TGA data of M^Vin^MQ copolymers.

M^Vin^MQ-X	T_5%_ [°C]		Coke [%]
	Air	Ar	
M^Vin^MQ-25	305	375	83.8
M^Vin^MQ-50	310	423	81.8
M^Vin^MQ-75	297	368	81.2
M^Vin^MQ-100	325	424	83.5

## Data Availability

The original contributions presented in this study are included in the article/Supplementary Material. Further inquiries can be directed to the corresponding author.

## Supplementary Materials

The following supporting information can be downloaded at https://www.mdpi.com/article/10.3390/polym18030315/s1: Figure S1: ^1^H NMR spectrum of (1) M^Vin^MQ-100 (1:2) in 4 h of reaction, (2) M^Vin^MQ-100 (1:2) in 6 h of reaction; Figure S2: IR spectra before and after blocking of M^Vin^MQ-25, M^Vin^MQ-75, and M^Vin^MQ-100 (1:2); Figure S3: DSC data of M^Vin^MQ-50, M^Vin^MQ-75, and M^Vin^MQ-100 (1:2).

## References

[1] Robeyns C., Picard L., Ganachaud F. (2018). Synthesis, characterization and modification of silicone resins: An “Augmented Review”. Prog. Org. Coat..

[2] Flagg D.H., McCarthy T.J. (2016). Rediscovering silicones: MQ copolymers. Macromolecules.

[3] Mironova M., Meshkov I., Shabeko A., Shutov V., Kulichikhin V., Tatarinova E. (2020). Rheological and Rheokinetic Properties of Compositions Based on a Butyl Rubber, an MQ Copolymer, and Polymethylsilsesquioxane. INEOS OPEN.

[4] Lewis L.N., Wengrovius J.H., Burnell T.B., Rich J.D. (1997). Powdered MQ resin—Platinum complexes and their use as silicone-soluble hydrosilylation cure catalysts. Chem. Mater..

[5] Zhu Y., Cao B., Zhang J., Zhai X., Guan D. (2020). Preparation and property study of organosilicon antisticking coatings. Adv. Mater. Sci. Eng..

[6] Sawvel A.M., Crowhurst J.C., Mason H.E., Oakdale J.S., Ruelas S., Eshelman H.V., Maxwell R.S. (2021). Spectroscopic signatures of MQ-resins in silicone elastomers. Macromolecules.

[7] Chen D., Chen F., Hu X., Zhang H., Yin X., Zhou Y. (2015). Thermal stability, mechanical and optical properties of novel addition cured PDMS composites with nano-silica sol and MQ silicone resin. Compos. Sci. Technol..

[8] Jochem H., Rejsek-Riba V., Maerten E., Remaury S., Solé S., Sierra G., Baceiredo A., Guillaumon O. (2013). Effects of 400 keV electrons flux on two space grade silicone rubbers. Mater. Chem. Phys..

[9] Kuo C.F.J., Chen J.B., Shih C.Y., Huang C.Y. (2014). Silicone resin synthesized by tetraethoxysilane and chlorotrimethylsilane through hydrolysis–condensation reaction. J. Appl. Polym. Sci..

[10] Venkatraman S., Gale R. (1998). Skin adhesives and skin adhesion: 1. Transdermal drug delivery systems. Biomaterials.

[11] Zhang Y., Zeng X., Lai X., Li H., Zhou Q., Huang X. (2017). Suppression effect and mechanism of amine-containing MQ silicone resin on the tracking and erosion resistance of silicone rubber. ACS Omega.

[12] Antosik A.K., Bednarczyk P., Czech Z. (2018). Aging of silicone pressure-sensitive adhesives. Polym. Bull..

[13] Xiang H., Ge J., Cheng S., Han H., Cui S. (2011). Synthesis and characterization of titania/MQ silicone resin hybrid nanocomposite via sol–gel process. J. Sol-Gel Sci. Technol..

[14] Shi X., Chen Z., Yang Y. (2014). Toughening of poly (l-lactide) with methyl MQ silicone resin. Eur. Polym. J..

[15] Jia P., Liu H., Liu Q., Cai X. (2016). Thermal degradation mechanism and flame retardancy of MQ silicone/epoxy resin composites. Polym. Degrad. Stab..

[16] Zheng J., Cai Y., Hu Y., Zhu J., Wei J., Ma Y., Wan J., Fan H. (2022). Bio-based epoxy functionalized MQ silicone resins: From synthesis to toughened epoxy composites with good mechanical properties, thermal resistance and transparency. Polym. Chem..

[17] Tatarinova E., Vasilenko N., Muzafarov A. (2017). Synthesis and properties of MQ copolymers: Current state of knowledge. Molecules.

[18] Voronina N., Meshkov I., Myakushev V., Laptinskaya T., Papkov V., Buzin M., Il’Ina M., Ozerin A., Muzafarov A. (2010). Hybrid organo-inorganic globular nanospecies: Transition from macromolecule to particle. J. Polym. Sci. Part A Polym. Chem..

[19] Liu Q., Dong H., Zhang Y., Wang E., Qu Z., Feng Q., Wu C. (2022). Preparation and Properties of α-Cyanoacryloyloxyethoxypropyl-functionalized MQ Silicone Resin. ChemistrySelect.

[20] Wang H., Zhu J., Gou H., Fang W., Fan H. (2025). Structure optimization of epoxy MQ silicone resin for excellent transparency, wear resistance, and anti-smudge coatings. React. Funct. Polym..

[21] Chen W., Zeng X., Lai X., Li H., Pan Z. (2018). Effect and mechanism of ureido-modified MQ silicone resin and platinum on tracking and erosion resistance of silicone rubber. Polym. Test..

[22] Borisov K.M., Kalinina A.A., Bokova E.S., Ilyina M.N., Cherkaev G.V., Tatarinova E.A., Milenin S.A., Bystrova A.V., Moeller M., Muzafarov A.M. (2022). Synthesis and properties of MQ resins with phenyl groups in monofunctional units. Mendeleev Commun..

[23] Egorova E., Vasilenko N., Demchenko N., Tatarinova E., Muzafarov A. (2009). Polycondensation of alkoxysilanes in an active medium as a versatile method for the preparation of polyorganosiloxanes. Dokl. Chem..

[24] Mironova M., Shandryuk G., Shabeko A., Meshkov I., Kulichikhin V., Muzafarov A. (2021). Kinetic Features of the Crosslinking Process for Compositions Based on Butyl Rubber and Dispersed Fillers. Polym. Sci. Ser. B.

[25] Cherepanov I., Trankina E., Frolova N., Sergienko N., Polshchikova N., Nelyubina Y.V. (2022). Synthesis of Disiloxanes and MQ Resins with Sydnonyl Substituents. Silicon.

[26] Mironova M., Meshkov I., Kulichikhin V., Muzafarov A. (2023). Effect of the chemical nature of MQ copolymers on the rheological properties of compositions on their basis. INEOS OPEN.

[27] Mironova M., Meshkov I., Shandryuk G., Kulichikhin V., Muzafarov A. (2024). Crosslinking Kinetics for Blends of Polyisoprene and MQ Copolymers. Polym. Sci. Ser. B.

[28] Meshkov I., Kalinina A., Kazakova V., Demchenko A. (2020). Densely cross-linked polysiloxane nanogels. INEOS OPEN.

[29] Sheima Y., Caspari P., Opris D.M. (2019). Artificial muscles: Dielectric elastomers responsive to low voltages. Macromol. Rapid Commun..

[30] Sheima Y., Yuts Y., Frauenrath H., Opris D.M. (2021). Polysiloxanes modified with different types and contents of polar groups: Synthesis, structure, and thermal and dielectric properties. Macromolecules.

[31] Krizhanovskiy I., Temnikov M., Drozdov F., Peregudov A., Anisimov A. (2023). Sequential hydrothiolation–hydrosilylation: A route to the creation of new organosilicon compounds with preset structures. React. Chem. Eng..

[32] Vinogradov S., Polivanov A., Chuprova E. (2019). Vinyl-functional MQ-resins. Plast. Massy.

[33] Ji J., Ge X., Liang W., Pang X., Liu R., Wen S., Sun J., Chen X., Ge J. (2019). Synthesis of High Molecular Weight Vinylphenyl-Con Taining MQ Silicone Resin via Hydrosilylation Reaction. Coatings.

[34] Ji J., Ge X., Pang X., Liu R., Wen S., Sun J., Liang W., Ge J., Chen X. (2019). Synthesis and characterization of room temperature vulcanized silicone rubber using methoxyl-capped MQ silicone resin as self-reinforced cross-linker. Polymers.

[35] Yang R., Dai Z., Kong Z., Ou J. (2024). Study on the thermal stability of TPU modified by MQ resin-filled silicone rubber. J. Polym. Res..

[36] Shang R., Wang L., Yin F., Farzaneh M. (2023). Investigation of MQ resin enhanced material used for on-site insulating bare conductors. IEEE Trans. Power Deliv..

[37] Huang Z., Wu J., Liu X., Ji H., He R., Liu R., Pimhataivoot P., Chen X. (2018). Versatile cascade esterification route to MQ Resins. ACS Omega.

[38] Czerwinski W.K. (1997). Improved increments for characterization of comonomer sequencing in binary copolymers. Polymer.

[39] Grubisic Z., Rempp P., Benoit H. (1967). A universal calibration for gel permeation chromatography. J. Polym. Sci. Part B Polym. Lett..

