# Supplementary material for: Synthesis of Vinyl-Containing MQ Copolymers in Active Medium
Source: Polymers (Basel). 2026 Jan 24;18(3):315. doi: 10.3390/polym18030315 (PMC12899343; doi:10.3390/polym18030315)
Supplement: Supplementary file 1 [file polymers-18-00315-s001.zip › polymers-4066059-supplementary.pdf]

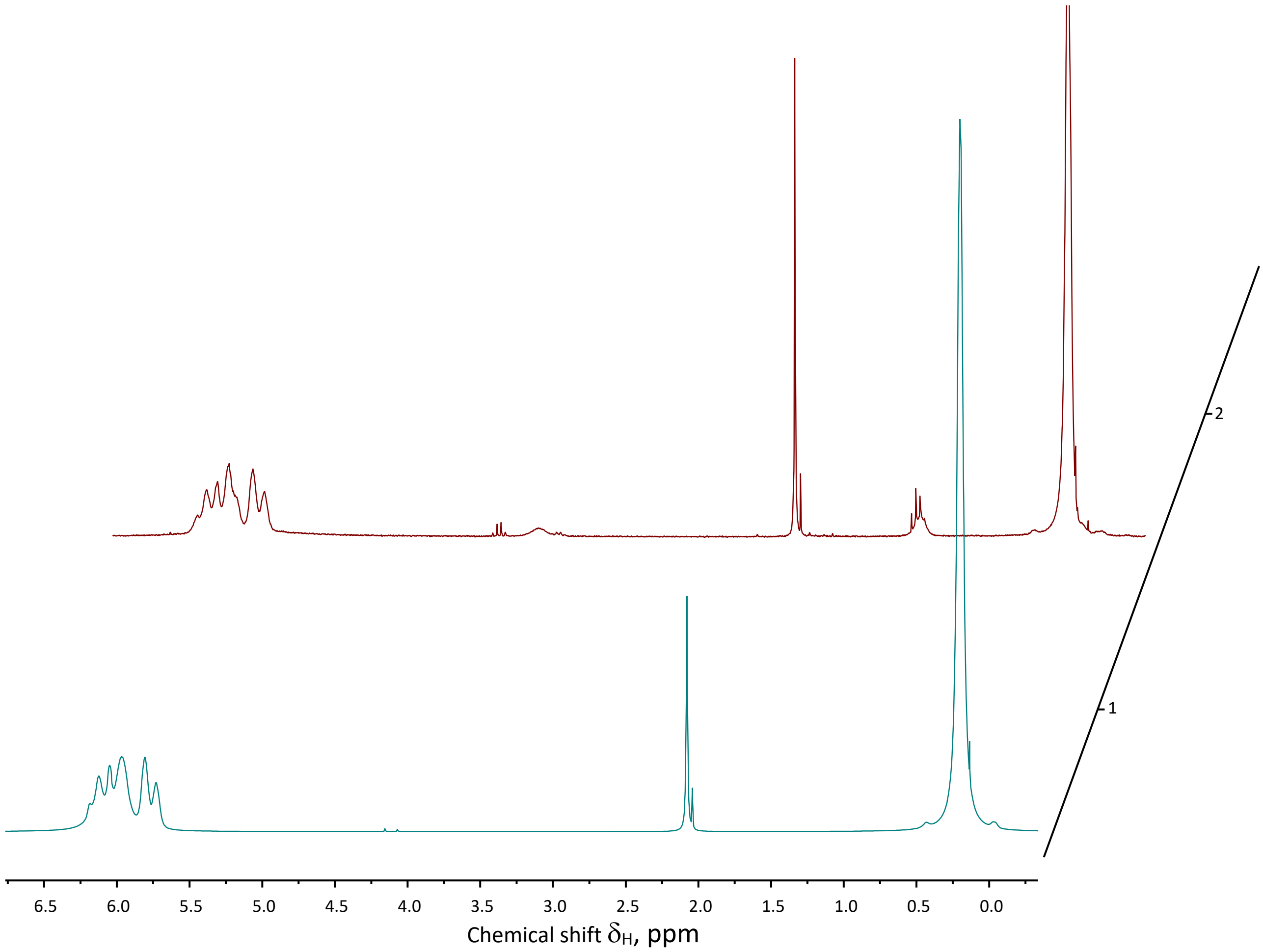

Figure S1. ^{1}H NMR spectrum of (1) M^{Vin}MQ-100 (1:2) in 4 h of reaction, (2) M^{Vin}MQ-100 (1:2) in 6 h of reaction

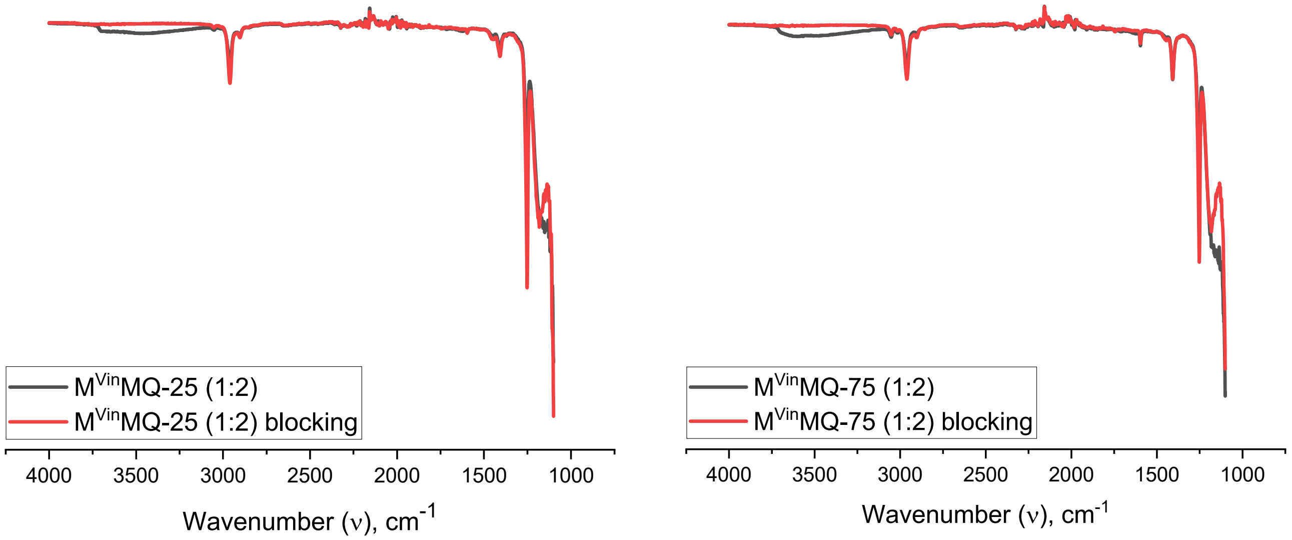

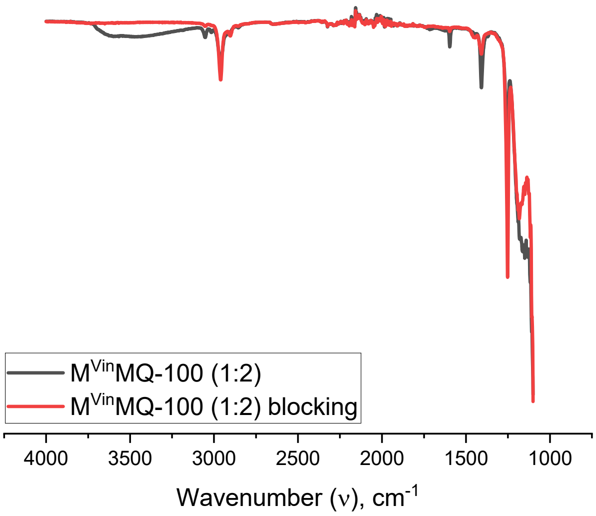

Figure S2. IR spectra before and after blocking of $M^{Vin}MQ$-25, $M^{Vin}MQ$-75 and $M^{Vin}MQ$-100 (1:2)

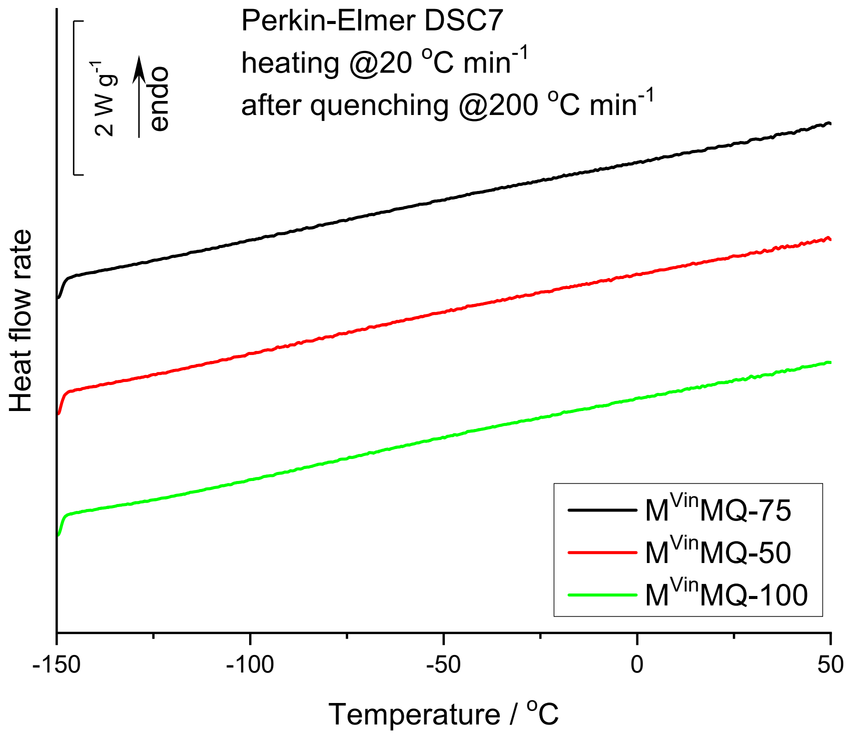

Figure S3. DSC data of $M^{Vin}MQ$-50, $M^{Vin}MQ$-75 and $M^{Vin}MQ$-100 (1:2)
